# Alpha-Calcitonin Gene Related Peptide: New Therapeutic Strategies for the Treatment and Prevention of Cardiovascular Disease and Migraine

**DOI:** 10.3389/fphys.2022.826122

**Published:** 2022-02-11

**Authors:** Ambrish Kumar, Maelee Williamson, Andrew Hess, Donald J. DiPette, Jay D. Potts

**Affiliations:** ^1^Department of Cell Biology and Anatomy, School of Medicine, University of South Carolina, Columbia, SC, United States; ^2^Department of Internal Medicine, School of Medicine, University of South Carolina, Columbia, SC, United States

**Keywords:** alpha-calcitonin gene-related peptide (α-CGRP), cardiovascular diseases, CGRP-agonist, CGRP-antagonist, heart failure, hypertension, migraine, neuropeptide

## Abstract

Alpha-calcitonin gene-related peptide (α-CGRP) is a vasodilator neuropeptide of the calcitonin gene family. Pharmacological and gene knock-out studies have established a significant role of α-CGRP in normal and pathophysiological states, particularly in cardiovascular disease and migraines. α-CGRP knock-out mice with transverse aortic constriction (TAC)-induced pressure-overload heart failure have higher mortality rates and exhibit higher levels of cardiac fibrosis, inflammation, oxidative stress, and cell death compared to the wild-type TAC-mice. However, administration of α-CGRP, either in its native- or modified-form, improves cardiac function at the pathophysiological level, and significantly protects the heart from the adverse effects of heart failure and hypertension. Similar cardioprotective effects of the peptide were demonstrated in pressure-overload heart failure mice when α-CGRP was delivered using an alginate microcapsules-based drug delivery system. In contrast to cardiovascular disease, an elevated level of α-CGRP causes migraine-related headaches, thus the use of α-CGRP antagonists that block the interaction of the peptide to its receptor are beneficial in reducing chronic and episodic migraine headaches. Currently, several α-CGRP antagonists are being used as migraine treatments or in clinical trials for migraine pain management. Overall, agonists and antagonists of α-CGRP are clinically relevant to treat and prevent cardiovascular disease and migraine pain, respectively. This review focuses on the pharmacological and therapeutic significance of α-CGRP-agonists and -antagonists in various diseases, particularly in cardiac diseases and migraine pain.

## Introduction

Alpha-calcitonin gene-related peptide (α-CGRP) is a regulatory neuropeptide, a potent vasodilator, and belongs to calcitonin gene family (Brain et al., 1985; Russell et al., 2014). It is predominantly produced in the central and peripheral nervous systems, specifically in the dorsal root ganglia (DRG) of Aδ and C sensory neurons. Another form of CGRP also exists, named β-CGRP, which is primarily synthesized in the enteric nervous system, pituitary gland, and immune cells (Steenbergh et al., 1985; Mulderry et al., 1988). On chromosome 11, α-CGRP is produced from the CALC-I gene while β-CGRP is produced from the CALC-II gene. Although, α- and β-CGRP are 94% homologous, α-CGRP is predominantly involved in hemodynamic actions, while β-CGRPs exert gastric effects in humans (Steenbergh et al., 1986). The present review is mainly focused on the pathophysiological function of α-CGRP in normal and disease conditions, particularly cardiovascular disease and migraine, and recent advances in developing CGRP-agonists and -antagonists.

## Alpha-Calcitonin Gene Related Peptide and Its Receptor

**FIGURE 1:**
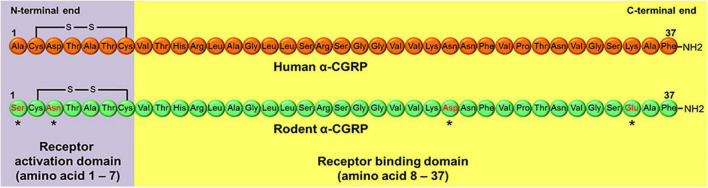
Peptide sequence of human and rodent α-CGRP. Rodent α-CGRP differs at four amino acids (*marked) with human α-CGRP. Amino acids 1–7 at N-terminal end of the peptide forms receptor activation domain, while amino acid region 8–37 consists of receptor binding domain.

α-CGRP is a peptide of 37 amino acids that contains a disulfide bond at amino acids 2 and 7, and a phenylalanine amide group at the carboxy-terminal end (Kumar et al., 2019a; Figure 1). The first seven amino acids at the N-terminus end form a ring-like structure (Maggi et al., 1990; Breeze et al., 1991). Deletion of disulfide bond at amino acids 2 and 7 prevents α-CGRP from exerting its physiological actions. A CGRP fragment devoid of first seven amino acids, a.k.a. CGRP_8–37_, can bind to the CGRP receptor, although its binding affinity for the receptor is 10-fold less than α-CGRP, and does not stimulate any physiologic response. CGRP_8–37_ acts as a competitive antagonist of the CGRP receptor (Chiba et al., 1989). Thus, the first seven amino acids play an essential role in α-CGRP’s high affinity binding for the CGRP receptor and are responsible for activating this receptor. Amino acid residues from 8 to 18 form an α-helix, while amino acids spanning from 19 to 27 form a β- or γ-twist (Rovero et al., 1992; Howitt et al., 2003). The C-terminal end of α-CGRP containing amino acids 28–37 interacts with N-terminus region of the CGRP receptor (Carpenter et al., 2001; Banerjee et al., 2006). Several biological and mutational studies have established that the N-terminal region of α-CGRP from amino acids 1–7 is responsible for receptor activation, while amino acids 8–37 are needed for binding affinity to the CGRP receptor (Watkins et al., 2013; Figure 1).

**FIGURE 2:**
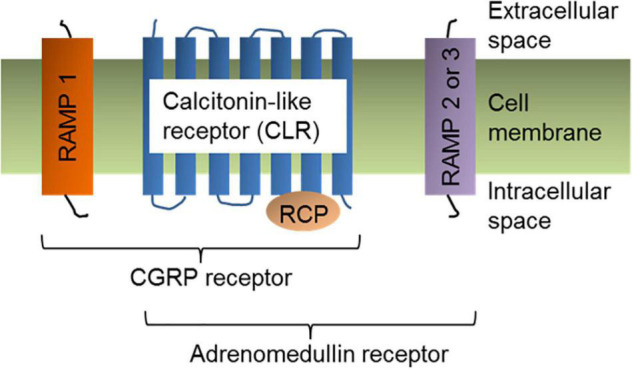
α-CGRP receptor structure. Cell-membrane bound α-CGRP receptor is a heterotrimer of three protein subunits: Calcitonin-like receptor (CLR), RAMP1, and RCP. Coupling of RAMP2 or RAMP3 with CLR and RCP subunits form adrenomedullin receptor.

The CGRP receptor is a heterotrimer of three protein subunits: (1)- Calcitonin receptor-like receptor (CLR), (2)- receptor activity modifying protein 1 (RAMP1), and (3)- receptor component protein (RCP) (Walker et al., 2010; Figure 2). All three protein components are essential to exhibit cellular functions of CGRP receptor. CLR is a member of the class B “secretin-like” family of the G-protein-coupled receptors (GPCR), which also contains the receptors for calcitonin, parathyroid hormone, vasoactive intestinal polypeptide, and pituitary adenylate cyclase activating polypeptide. The ligand-binding CLR is a 461 amino acid protein that contains seven transmembrane-spanning domains, an extracellular N-terminus, and a cytosolic C-terminus (Aiyar et al., 1996). To initiate the signaling cascade, α-CGRP binds to the extracellular N-terminus of CLR. RAMP1 is a member of the RAMP protein family, which also includes RAMP2 and RAMP3 (Hay and Pioszak, 2016; Serafin et al., 2020). The RAMP family shares a similar structural motif even though their amino acid sequences are <30% homologous with one another. RAMP1 is specific to the CGRP receptor, while RAMP2 and RAMP3 are part of the adrenomedullin (AM) receptors (McLatchie et al., 1998; Figure 2). RAMP1 consists of one transmembrane-spanning domain with a long extracellular N-terminal domain (∼100 amino acids) and a short intracellular C-terminus (∼10 amino acids) (Steiner et al., 2002; Udawela et al., 2006; Qi and Hay, 2010). In order for CLR to be transported from the endoplasmic reticulum to the plasma membrane, it must be heterodimerized with RAMP1 as a mature glycoprotein (Hilairet et al., 2001). The heterodimeric complex of CLR and RAMP1 is stabilized via non-covalent interactions between the two proteins. Production of RAMP1 in the absence of CLR inhibits RAMP1 transport to the plasma membrane, resulting in RAMP1 accumulation in the Golgi apparatus as a disulfide-linked homodimer. In RAMP1-KO mice, an increase in BP (but no change in heart rate) was observed suggesting that deletion of RAMP1 disrupted CGRP-mediated cellular signaling (Tsujikawa et al., 2007). Receptor component protein (RCP) is a small (∼17 kDa) cytosolic protein that ionically binds to CLR and is required for G protein coupling (Evans et al., 2000; Egea and Dickerson, 2012). Experiments using RCP-depleted NIH3T3 cells concluded that although loss of RCP levels had no effect between the binding affinity of α-CGRP and CLR, these cells were unable to initiate the CGRP signaling cascade to create a physiologic response due to decreased intracellular cyclic adenosine monophosphate (cAMP) levels (Evans et al., 2000). Therefore, RCP serves as the connection between CLR and the intracellular G protein-mediated signaling pathway, which triggers production of cAMP (Routledge et al., 2020).

The expression of CGRP receptor is widely reported within the central and peripheral nervous system, and cardiovascular system. Molecular methods like radioligand binding assay, immunohistochemistry, and Northern blot analysis were employed to determine the localization of CGRP receptor in different tissues and organs. Using radiolabelled ^125^I-CGRP in radioligand binding assays, the specific binding site for the CGRP ligand has been shown in the rat cardiovascular system, human and rat brain, central and peripheral tissues including bronchial and pulmonary blood vessels of human and guinea pig, and the smooth muscle layer of arteries and arterioles of the human urinary bladder (Inagaki et al., 1986; Sexton et al., 1986; Mak and Barnes, 1988; Wimalawansa and MacIntyre, 1988; van Rossum et al., 1997; Burcher et al., 2000). In the cardiovascular system and visceral organs, CGRP-binding sites were detected in the right atrium, and within the intima and media layers of blood vessels, such as the mesenteric artery, aorta, carotid arteries, renal arteries, pulmonary arteries and veins, spleen, lung, and liver (Nakamuta et al., 1986; Wimalawansa and MacIntyre, 1988; McCormack et al., 1989). The mRNA transcripts of CLR (by RT-PCR assay) and RAMP1 (by Northern blot analysis) were detected in myocyte and non-myocyte cells prepared from cardiac ventricles of 1-day-old rat pups (Tomoda et al., 2001). In addition, using immunohistochemistry the expression of CGRP receptor components (CLR and RAMP1) in arterial blood vessels and nerve fibers present in the rodent cranial dura mater, human trigeminal ganglion, myelinated A-fiber axons, and rat cerebellar Purkinje cells were detected (Lennerz et al., 2008; Eftekhari et al., 2010, 2013; Edvinsson et al., 2011). In the rat brain, the immunoreactivity for CLR1 and RAMP1 was detected in the neuronal processes of the cerebral cortex, hippocampus, cerebellum, thalamic and hypothalamic nuclei, and brainstem nuclei (Warfvinge and Edvinsson, 2019). Using human subcutaneous arteries, Edvinsson et al. (2014), detected the immunoreactivity of CLR and RAMP1 in the endothelial and smooth muscle cells, and to a minor extent in the vascular adventitia (Edvinsson et al., 2014). Eftekhari and Edvinsson (2011) performed immunohistochemistry for CGRP, CLR and RAMP1 and compared their expression in the human and rat spinal trigeminal nucleus (STN) and the C1 region of the spinal cord (Eftekhari and Edvinsson, 2011). Immunohistochemistry data demonstrated that CLR and RAMP1 positive fibers were present in the human spinal trigeminal tract region. CLR and RAMP1 were co-localized in this region, but no co-localization of CGRP and CLR or CGRP and RAMP1 was observed. In rat STN, CLR and RAMP1 (but not CGRP and CLR or CGRP and RAMP1) were co-localized in the spinal trigeminal tract region, in both fiber bundles and fibers spanning from the spinal trigeminal tract. In human C1, CLR and RAMP1 signals were detected within laminae I and II, but no co-localization of these receptor components was observed. However, in rat C1, CLR and RAMP1 were co-localized in the laminae I and II region, but no co-localization of CGRP and CLR or CGRP and RAMP1 was observed. In contrast to Eftekhari and Edvinsson (2011) study, one study reported partial co-localization of CGRP and its receptor component CLR and RAMP1 in the rat superficial laminae (Lennerz et al., 2008). The discrepancy in the detection of CGRP, CLR, and RAMP1 in both studies might be due the difference in the recognition sites of the antibodies used and change in methods of the tissue processing. Miller et al. (2016), raised antibodies against a fusion protein of the extracellular domains of RAMP1 and CLR that comprise the CGRP binding site of the CGRP receptor. Using these antibodies, researchers performed immunohistochemistry and confirmed the expression of CGRP receptor in human vascular smooth muscle cells of dural meningeal arteries and neurons in the trigeminal ganglion (Miller et al., 2016). Immunohistology method further confirmed expression of CLR and RAMP1 in human cerebral vasculature particularly in middle meningeal, middle cerebral, superficial temporal, and pial vessels (Oliver et al., 2002). These studies suggested that although immunohistochemistry is a powerful tool to detect regional distribution of proteins in tissue, it has several limitations as well. A variety of antibodies raised against CGRP and CGRP receptor components are commercially available, however, considerations such as consistency in tissue processing, use of validated antibodies specific for the histochemistry, and species specificity of these antibodies are needed while performing immunohistochemistry to detect CGRP and its receptor in the tissue.

## Synthesis and Release of Alpha-Calcitonin Gene Related Peptide

**FIGURE 3:**
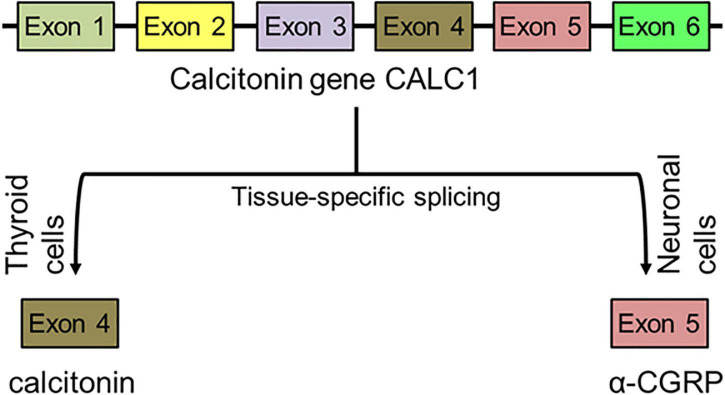
Synthesis of peptide α-CGRP. After tissue-specific alternative splicing of calcitonin gene CALC1, α-CGRP is synthesized in the neuronal cells while calcitonin is formed in the thyroid cells.

**FIGURE 4:**
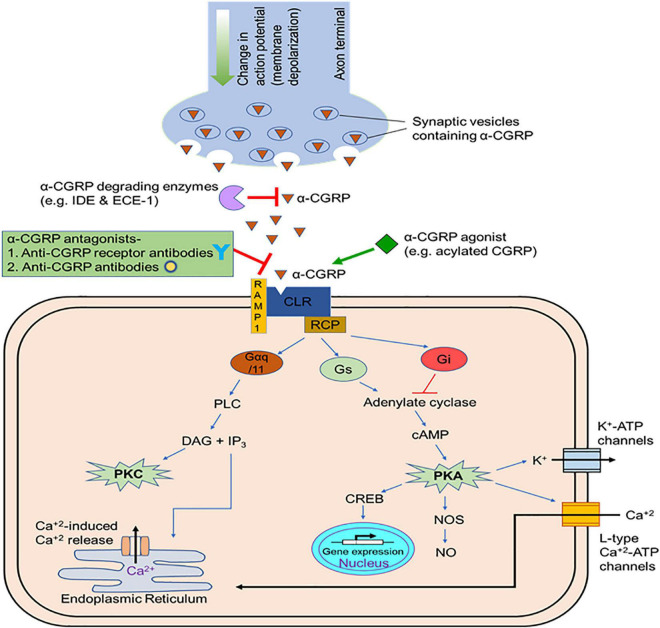
Release and mode of action of α-CGRP. After synthesis, α-CGRP remains stored in vehicles at the terminal ends of the nerves. As nerve depolarize, α-CGRP is released form the nerve terminals through the exocytosis process and interacts to its receptor and elicits its cellular effects mainly via the generation of intracellular cAMP. In the circulation, endopeptidases (insulin degrading enzyme, IDE, and endothelin converting enzyme-1, ECE-1) degrades α-CGRP. CGRP-agonists with increased self-life and bioactivity prolong the cellular activity of α-CGRP and might be beneficial to use as therapeutic agents in cardiac diseases. Several CGRP-antagonists that block interaction of α-CGRP to its receptor and, thus, inhibit downstream signaling pathways are employed to treat migraine pain.

The α-CGRP peptide is created via tissue-specific alternative splicing of the pre-mRNA transcript of the calcitonin gene CALC-I on chromosome 11 (Amara et al., 1982). Thyroid C cells produce calcitonin by splicing out exons 5 and 6 from the CALC-I gene transcript, while sensory neurons form α-CGRP via removal of exon 4 from the CALC-I gene pre-mRNA product (Figure 3). To produce mature α-CGRP, it is first translated as a 121 amino acid pro-hormone and then cleaved into a final 37 amino acid polypeptide (Rosenfeld et al., 1983). α-CGRP is primarily stored and released from Aδ- and C-fiber sensory neurons of the afferent nervous system, though it is also distributed in smooth muscle of blood vessels and in regions of the central nervous system (Gibson et al., 1984; Uddman et al., 1986; Kee et al., 2018). α-CGRP synthesis occurs almost exclusively in the dorsal root ganglia (DRG) of sensory neurons, however, endothelial cells and certain immune cells, such as lymphocytes and monocytes, also have the ability to produce the peptide (Doi et al., 2001; Bracci-Laudiero et al., 2002; Wang et al., 2002; Linscheid et al., 2004). Additionally, the trigeminal ganglion releases α-CGRP to regulate cerebral vascular tone, which contributes to the pathology of certain migraines (McCulloch et al., 1986). Aδ- and C-fiber sensory neurons release α-CGRP when stimulated by mechanical, thermal, and chemical stimuli, but because of their efferent function, they can also excrete α-CGRP without any stimulation. It is hypothesized that the efferent release of α-CGRP from these neurons plays an important role in blood flow regulation, as circulating α-CGRP levels are attributable to leakage of the peptide from the synaptic cleft after its release. Once α-CGRP is generated, it is stored in neuronal vesicles with substance P (SP) in the axon terminals until axonal depolarization and calcium-dependent exocytosis trigger its release (Gibbins et al., 1985; Matteoli et al., 1988; Figure 4). In motor neurons, α-CGRP is stored and released with acetylcholine (Ach), which led some to hypothesize that α-CGRP plays a role in Ach receptor synthesis (New and Mudge, 1986; Csillik et al., 1993).

α-CGRP is released in response to high levels of angiotensin-II (Ang-II) and norepinephrine to combat the potent vasoconstrictive actions of these compounds, which is why α-CGRP agonists are likely beneficial in hypertensive patients (Supowit et al., 1995b). Capsaicin, nerve growth factor (NGF), brain-derived neurotropic factor (BDNF), low pH, bradykinins, prostaglandins, tissue inflammation, and the renin angiotensin aldosterone system (RAAS) also promote α-CGRP excretion from DRG sensory neurons (Lindsay et al., 1989; Donnerer and Stein, 1992; Andreeva and Rang, 1993; Strecker et al., 2006; Salio et al., 2007; Supowit et al., 2011). Additionally, α-CGRP synthesis increases in the presence of neuronal damage (Donnerer et al., 1992). It is thought that macrophages and keratinocytes excrete NGF in a paracrine-like manner when exposed to inflammation in order to stimulate nearby sensory neurons to release α-CGRP. One study demonstrated that addition of NGF to adult DRG neurons promotes α-CGRP expression through activation of the Ras/Raf/MEK-1/p42/p44 MAPK pathway (Freeland et al., 2000). Another study illustrated that NGF intraperitoneal injections in spontaneously hypertensive rats (SHRs) induced α-CGRP production and release in DRG sensory neurons (Supowit et al., 2001). Stimulation of the transient receptor potential vanilloid 1 (TRPV1) receptor, a type of non-selective cation channel in the TRP family found on sensory nerve axon terminals, prompts α-CGRP release when exposed to noxious heat, capsaicin, and acidic environments (Kichko and Reeh, 2009; Meng et al., 2009; Dux et al., 2020). It has been observed that phenotypically TRPV1-KO mice are not hypertensive, however, these KO mice, after undergoing TAC procedure, exhibit increased LV hypertrophy and fibrosis compared to their wild-type counterparts (Marshall et al., 2013; Zhong et al., 2018). Transient receptor potential ankyrin 1 (TRPA1), another member of the TRP family, induces α-CGRP excretion when exposed to noxious cold, reactive oxygen species (ROS), and cellular stress (Pozsgai et al., 2012; Wang et al., 2019; Gebhardt et al., 2020).

A CGRP-KO mouse strain, where both CGRP and calcitonin genes were deleted, showed an increase in basal BP (Gangula et al., 2000), while no difference in heart rate and BP was observed in another CGRP-KO mouse strain (Lu et al., 1999). Moreover, compared with their WT-counterparts, α-CGRP mice exhibit a higher extent of cardiac fibrosis and inflammation when undergoing TAC-induced pressure overload, and their renal and cardiovascular systems are more susceptible to hypertension-induced end-organ damage (Supowit et al., 2005; Li et al., 2013).

## Therapeutic Benefits of Alpha-Calcitonin Gene Related Peptide and Its Analogs in Cardiovascular Disease

The effects of α-CGRP on the cardiovascular system are well-documented: it induces positive chronotropic, ionotropic, and hypertrophic effects on the heart, it mediates blood flow, and it is the most potent vasodilator known to date (Fisher et al., 1983; Brain et al., 1985; Franco-Cereceda and Lundberg, 1985; Struthers et al., 1986; Gennari et al., 1990; Bell et al., 1995; Al-Rubaiee et al., 2013). The positive ionotropic and chronotropic actions of α-CGRP are attributed to reflexive sympathetic activity and direct stimulation of α-CGRP on cardiomyocytes to oppose the hypotensive effects from vasodilation. Specifically, α-CGRP stimulation of cardiomyocytes triggers positive ionotropic effects via the cAMP/PKA or the PKC intracellular messenger pathways. An *in vitro* study of guinea pig atria found that exposure to capsaicin, a TRPV1 agonist, triggered α-CGRP release and produced positive ionotropic and chronotropic effects in the cardiac cells, but these results were diminished when hearts were infused with capsaicin to desensitize the nerves and induce tachyphylaxis (Lundberg et al., 1984). The vasodilatory capabilities of α-CGRP are ∼1,000 times more potent than acetylcholine, substance P, and 5-hydroxytriptamine, and 10 times more potent than the most powerful vasodilatory prostaglandins (Brain et al., 1985). Additionally, α-CGRP-induced vasodilation persists longer than other vasodilators. Injection of 15 pmol of α-CGRP intradermally in humans augments local blood flow and causes erythema formation of the skin that lasts 5–6 h (Brain et al., 1985). One study carried out by DiPette et al. (1989) demonstrated that a bolus intravenous injection of α-CGRP at doses of 22, 65, 220, and 2,200 pmol in conscious rats reduced mean blood pressure in a dose dependent manner (DiPette et al., 1989). Because of its potency and sustainability, α-CGRP stimulates vasodilation of various vascular beds, including the coronary, cerebral, and renal vessels, and systemic infusion reduces blood pressure in normotensive and hypertensive species (DiPette et al., 1989; Dubois-Rande et al., 1992; Gulbenkian et al., 1993). The vasodilatory actions of α-CGRP are most prominent in small peripheral arteries, as opposed to large vessels, respectively. It has been shown in the rat isolated mesenteric resistance arteries that α-CGRP is used to combat the vasoconstrictive effects of endothelin via interaction with the CGRP receptor and a G_βγ_ -coupled protein (Meens et al., 2011, 2012).

α-CGRP can stimulate vasodilation of peripheral arteries using either the nitric oxide (NO)-/endothelium-independent pathway or the NO-/endothelium-dependent pathway. However, in most blood vessels, vasodilatation occurs via the NO-/endothelium-independent route (Russell et al., 2014; Kumar et al., 2019b). To initiate the NO-/endothelium-independent pathway, α-CGRP first binds to the CGRP receptor on a vascular smooth muscle cell, which stimulates Gα_*s*_ to activate adenylate cyclase. Once adenylate cyclase is stimulated, it will synthesize cAMP followed by activation of protein kinase A (PKA). The activated PKA subsequentially induces K^+^-ATP channels to open, causing smooth muscle relaxation and vasodilation. Administration of glibenclamide, a K^+^-ATP channel inhibitor, blocks α-CGRP-induced hyperpolarization, and therefore vasodilation, of vascular smooth muscle cells (Edvinsson et al., 1985; Nelson et al., 1990).

There are several reports showing that vasodilation of the rat aorta and pulmonary arteries, and human internal mammary arteries are regulated via the NO-/endothelium-dependent pathway. One study found that delivery of NO synthase (NOS) inhibitors in the rat aorta weakened the vasodilatory capability of α-CGRP (Gray and Marshall, 1992b). Another study demonstrated that treatment of rat aortas with human α-CGRP augmented intracellular cAMP and cGMP concentrations, and induced vasodilation. This occurred only when the endothelium was intact, further supporting the idea that these vessels are NO-/endothelium-dependent (Gray and Marshall, 1992a).

Several studies have highlighted the importance of α-CGRP as a cardioprotective molecule. Specifically, α-CGRP prevents pathologies such as hypertension, myocardial infarctions, heart failure, and ischemia from damaging cardiac cells through vasodilation and inhibition of oxidative stress, inflammation, and apoptosis. The role of α-CGRP in normal and pathophysiological states of these diseases is disused below.

### Hypertension

Plasma levels of α-CGRP are elevated in hypertensive patients with primary aldosteronism or on a high-salt diet, but are decreased in patients with secondary hypertension who have undergone an adrenalectomy, suggesting that α-CGRP might act as a compensatory mechanism: first the peptide is released to oppose the effects of high blood pressure, but later CGRP synthesis and/or release may become inhibited as the disorder advances (Masuda et al., 1992). Several models of hypertension have revealed that expression of α-CGRP and its receptor increased in these pathological states (Kawasaki et al., 1990; Supowit et al., 1995a; Li and Wang, 2005). In a number of experimental hypertension models such as deoxycorticosterone (DOC)-salt, subtotal nephrectomy (SN)-salt, the two-kidney one-clip (2K1C), N-nitro-L-arginine methyl ester (L-NAME)-induced hypertension during pregnancy, and chronic hypoxic pulmonary hypertension, α-CGRP blunts blood pressure elevations as a compensatory mechanism (Tjen et al., 1992; Gangula et al., 1997; Supowit et al., 1997, 1998). Depending on the model of hypertension, neuronal levels of α-CGRP are regulated differently. For example, in hypertensive rats induced by either the DOC-salt or the 2K1C model, levels of immunoreactive CGRP in the spinal cord and α-CGRP mRNA in DRG were elevated in order to stimulate vasodilation and combat the developing hypertension (Supowit et al., 1995a,^1997^, ^1998^). Administration of capsaicin or the CGRP receptor antagonist, CGRP_8–37_, in these rats increased the mean arterial pressure (MAP), further supporting the idea that the high α-CGRP levels attenuated the hypertension. Delivery of CGRP_8–37_ in SN-induced hypertensive rats and L-NAME-induced hypertension during pregnancy also augmented the hypertensive levels (Gangula et al., 1997). In contrast to the DOC-salt model, immunoreactive CGRP concentrations in laminae I and II of the spinal cord and α-CGRP mRNA levels in the DRG were reduced in spontaneously hypertensive rats (SHRs) (Supowit et al., 1993). Further, pre-treatment administration of capsaicin or CGRP_8–37_ had no effect on blood pressure, indicating that the lack of α-CGRP contributes to the development of high blood pressure due to loss of vasodilatory control.

One study demonstrated that Ang-II-induced hypertensive mice displayed higher amounts of ROS, inflammation, and apoptotic cell death in their hearts and kidneys. Additionally, they showed reduced cardiac function due to Ang-II upregulation of oxidative stress response proteins and downregulation of the anti-oxidative enzyme glutathione peroxidase-1, resulting in disruption of endothelial cell function and vascular hypertrophy (Aubdool et al., 2017). Furthermore, Ang-II induces RAMP1 expression in the cardiovascular system. Delivery of acylated α-CGRP, an α-CGRP agonist, in mice significantly reduced the adverse effects created by Ang-II. A similar study using α-CGRP KO mice with hypertension via Ang-II administration, led to aortic hypertrophy and reduced eNOS expression (Smillie et al., 2014). Therefore, α-CGRP appears to delay the onset and development of hypertension through cardioprotective mechanisms.

A study comparing α-CGRP KO mice to WT mice in a DOC-salt model revealed that the KO mice developed greater amounts of cellular damage and oxidative stress in the cardiovascular and renal systems and had elevated concentrations of pro-inflammatory cytokines and chemokines than the DOC-salt WT mice (Supowit et al., 2005). Baseline blood pressure of α-CGRP KO mice was significantly elevated compared to WT counterparts, indicating that α-CGRP plays a role in preventing hypertension. Therefore, lack of α-CGRP increases the likelihood of hypertension-induced damage to the heart and kidneys. Another basis for α-CGRP KO mice to develop high blood pressure is that they have greater activity of the RAAS, which is a well-known mechanism involved in the development of hypertension (Li et al., 2004). α-CGRP opposes the hypertensive effects of RAAS through vasodilation and by inhibiting Ang-II induced aldosterone secretion. Electric field stimulation of the mesenteric arteries in SHRs triggers a vasoconstrictive response, but the vasodilatory response to α-CGRP was augmented by aldosterone, suggesting that α-CGRP controls blood pressure homeostasis by interacting with the RAAS (Balfagon et al., 2004). Delivery of sub-depressor doses of α-CGRP for 6 days in hypertensive rats induced by Ang-II or norepinephrine decreased blood pressure significantly (Fujioka et al., 1991). Much like α-CGRP KO mice, RAMP1 KO mice develop high blood pressure (Tsujikawa et al., 2007). However, even with exogenous α-CGRP administration, RAMP1 KO mice are hypertensive because α-CGRP is unable to activate its signaling cascade and therefore, vasodilation is unachievable. On the other hand, upregulation of RAMP1 in Ang-II-induced hypertensive mice enhanced the ability of α-CGRP to reduce blood pressure (Sabharwal et al., 2010). Additionally, because the baroreflex sensitivity of RAMP1 transgenic mice was augmented, resulting in a lower blood pressure, suggesting that α-CGRP modulates the baroreflex response to help reduce blood pressure.

α-CGRP is also protective against pulmonary hypertension, likely as the result of the high expression in the lungs and dilatation of pulmonary arteries (Bivalacqua et al., 2002). α-CGRP specifically acts to blunt the effects of hypoxia-induced tissue remodeling that occurs in pulmonary hypertension. Rats affected by pulmonary hypertension have reduced plasma levels of α-CGRP, and these effects are exacerbated by CGRP_8–37_ infusion (Tjen et al., 1992). Administration of α-CGRP to these rats attenuated the effects of pulmonary hypertension. One study carried out using left-to-right shunt-induced pulmonary hypertensive rats demonstrated that intravenous injection of endothelial progenitor cells modified to secrete α-CGRP reduced the severity of the disease and prevented adverse vascular remodeling (Zhao et al., 2007). These studies demonstrated that α-CGRP is a potent vasopressor and is a potential therapeutic peptide to treat patients suffering from high blood pressure.

### Ischemia/Reperfusion Injury

The protective function of α-CGRP against ischemia/reperfusion (I/R) injury has been reported in human and a numerous animal models. α-CGRP KO mice subjected to 30-min ischemic episodes followed by reperfusion to stimulate I/R injury experienced significant deterioration in cardiac function compared to WT mice who underwent the same procedure (Huang et al., 2008). Sensory Aδ- and C-fibers excrete α-CGRP as a cardioprotective mechanism after a cardiac I/R injury. For example, levels of circulating α-CGRP increase in humans following an acute myocardial infarction and isolated hearts of guinea pigs and rats release more α-CGRP after experiencing myocardial ischemia (Franco-Cereceda, 1988; Mair et al., 1990; Lechleitner et al., 1992). During these pathological events, stimuli such as bradykinin, prostaglandins, and low pH activate TRPV1 receptors to induce α-CGRP release. An increased CGRP-immunoreactivity has been reported in principal ganglionic neurons and perineuronal nets in the human stellate ganglia following acute myocardial infarction (Roudenok et al., 2001). Other investigations have revealed that α-CGRP also plays a role in cardiac and remote preconditioning, in which CGRP infusion reduces the cardiac damage caused by an ischemic event in a rat model of I/R injury (Wolfrum et al., 2005). When Langendorff-perfused rat hearts were administered exogenous α-CGRP, the peptide imitated the beneficial preconditioning effects induced by transient ischemia and resulted in increased endogenous α-CGRP release (Wu et al., 2001). Conversely, infusion of the CGRP receptor antagonist BIBN4096BS inhibited all cardioprotective effects of α-CGRP and preconditioning, suggesting that α-CGRP is involved in the physiologic response to cardiac I/R injuries. BIBN4096BS or olcegepant (developed by Boehringer Ingelheim Pharmaceuticals) is a small molecule non-peptide antagonist that exhibits greater antagonism to the human CGRP receptor when compared to rodent CGRP-receptor, and does not show affinity for adrenomedullin receptors (AM1 or AM2 receptor) (Doods et al., 2000; Hay et al., 2003; Arulmani et al., 2004). In addition to the CGRP-receptor, BIBN4096BS is reported to block the binding of CGRP to another distinct CGRP-responsive receptor, the amylin subtype 1 receptor known as the AMY1 receptor, however, BIBN4096BS has higher binding affinity to the CGRP receptor than to the AMY1 receptor and it is a relatively selective antagonist to the CGRP receptor (Hay et al., 2006; Walker et al., 2018). A series of *in vitro* receptor assays carried out in Cos7 cells transfected with CGRP receptor or AMY1 receptor and in cultured rat trigeminal ganglia neurons demonstrated that BIBN4096BS treatment significantly inhibited α-CGRP-stimulated phosphorylation of cAMP response element-binding protein (CREB) and the accumulation of cAMP in these cells (Walker et al., 2018). A comparison study in the transfected Cos7 cells showed that BIBN4096BS was ∼132-fold more potent at the CGRP receptor than the AMY1 receptor in blocking α-CGRP-stimulated cAMP accumulation and ∼26-fold more potent at the CGRP receptor than the AMY1 receptor in blocking α-CGRP-stimulated CREB phosphorylation (Walker et al., 2018). Moreover, the antagonist selectivity of this molecule has been shown to be pathway-dependent as BIBN4096BS inhibited α-CGRP-stimulated CREB phosphorylation to a greater extent compared to cAMP accumulation in rat trigeminal ganglia neurons and AMY1 receptor transfected Cos7 cells (but not in the CGRP receptor transfected Cos7 cells) (Walker et al., 2018). The AMY1 receptor is a complex of the calcitonin receptor (CTR) and RAMP1 subunit (CTR-RAMP1), and it is responsive to both CGRP and amylin peptides (Muff et al., 1999; Walker et al., 2015; Hay and Walker, 2017). As RAMP1 is a common component in the CGRP receptor and AMY1 receptor, it is thought that binding of BIBN4096BS to RAMP1 might be involved in the antagonism of BIBN4096BS (Mallee et al., 2002). Due to poor oral bioavailability, BIBN4096BS is not considered a viable choice as a therapeutic molecule where inhibition of CGRP-receptor activation is needed, e.g., migraine headache. Adenoviral transfection of cardiac tissue with α-CGRP, in diabetic rodents, increased the ischemic preconditioning response, which is typically reduced in these models (Zheng et al., 2012). Zheng et al. (2012) showed that streptozotocin-induced diabetic mice who underwent ischemia (30 min) and reperfusion (24 h) had increased myocardial infarct size and higher plasma level of lactate dehydrogenase (LDH) compared to their non-diabetic counterparts. However, intramyocardial injection of adenovirus encoding the CGRP gene reduced myocardial infarct size and plasma LDH level in both non-diabetic and diabetic mice (Zheng et al., 2012). α-CGRP has been shown to prevent I/R damage in other tissues such as the intestines, kidneys, and brain (Song et al., 2009; Liu et al., 2011; Lu et al., 2017). When central organs, like the heart and liver, are exposed to ischemia, α-CGRP release may serve to both prevent damage to these organs and protect other organ systems, such as the intestines, from subsequent injury via reflexive neuronal release. In one study, Luo et al. (2016) demonstrated that following the intestinal ischemia or intestinal reperfusion phase in rats, the level of caspase-3 protein (an apoptotic cell death marker) increased. CGRP pretreatment reduced iNOS and caspase-3 levels, and protected against intestinal I/R injury (Luo et al., 2016).

Finally, administration of α-CGRP following a myocardial I/R injury can significantly reverse the developing cardiac dysfunction. In patients with stable angina pectoris, exogenous α-CGRP postponed the onset of myocardial ischemia (Mair et al., 1990). In total, these findings demonstrate that depletion of α-CGRP increases the risk of developing an I/R injury, augments cardiac damage, and delays the recovery process following myocardial ischemia. These results demonstrated that α-CGRP is protective against ischemia-reperfusion injury.

### Heart Failure

α-CGRP-producing sensory nerves are found in the perivascular layer of coronary vessels, in the cardiac conduction system, and in myocardium of the ventricles (Gulbenkian et al., 1993). When blood pressure rises or there is a potential of damage to the heart, such as ischemia or oxidative stress, these neurons release α-CGRP to maintain cardiovascular homeostasis. Exogenous administration of α-CGRP enhances cardiac function in humans by stimulating catecholamine release to induce positive ionotropic effects and by increasing stroke volume through reducing afterload via vasodilation (Tortorella et al., 2001). Additionally, α-CGRP infusion in patients with heart disease has been shown to enhance blood flow through vasodilation (Gennari et al., 1990; Shekhar et al., 1991). For these reasons, α-CGRP protects the heart against adverse myocardial remodeling and dysfunction from hypertension and heart failure.

**FIGURE 5:**
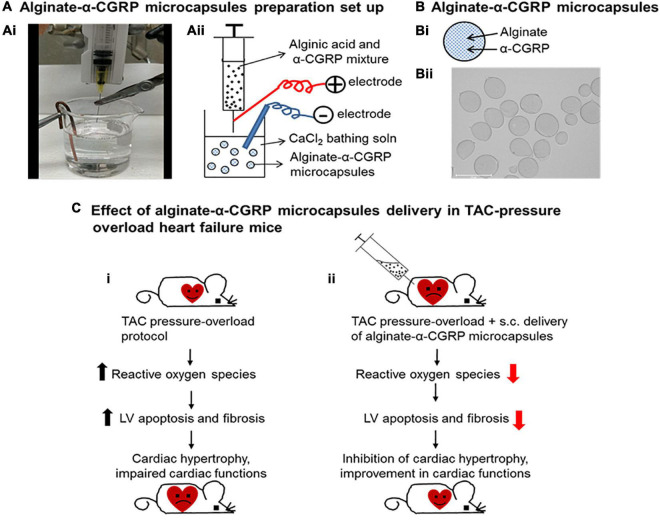
Alginate-α-CGRP microcapsules as a peptide delivery system in heart failure mice. Laboratory set up **(Ai)** and schematic presentation **(Aii)** showing encapsulation of α-CGRP in alginate microcapsules using an electrospray method. **(B)** Schematic picture showing α-CGRP encapsulated alginate microcapsule **(Bi)**. Using an electrospray method, alginate-α-CGRP microcapsules were prepared and imaged **(Bii)**. **(C)** Schematic representation of cardio-protective effect of alginate-α-CGRP microcapsules against heart failure in mice. Transverse aortic constriction (TAC) procedure was performed in wild-type mice. TAC-induced pressure overload stimulated excessive reactive oxygen species generation and induced apoptotic cell death and fibrosis in mice left ventricles (LV). As a result, hearts got enlarged and cardiac functions impaired in the TAC-mice **(Ci)**. Subcutaneous administration of alginate-α-CGRP microcapsules in TAC-mice attenuated increased levels of TAC-induced LV oxidative stress, apoptosis, and fibrosis that, in turn, inhibited cardiac hypertrophy and improved cardiac functions in the TAC-mice **(Cii)**.

Although patients with heart failure have higher circulating levels of α-CGRP during the initial and middle stages, these levels rapidly fall during the later stages of heart failure as functionality declines (Dubois-Rande et al., 1992). Rats induced with pressure-overload heart failure via constriction of their ascending aorta expressed higher levels of RAMP1 mRNA and its subsequent protein in the cardiomyocytes of their atria and ventricles, indicating that more CGRP receptors were available (Cueille et al., 2002). Another study using rats with isoprenaline-induced heart failure found that delivery of rutaecarpine, a TRPV1 agonist that stimulates α-CGRP release, attenuated cardiac hypertrophy and apoptosis of cardiomyocytes (Li et al., 2010). Pre-treatment capsaicin infusion to these rats diminished the results, further indicating that α-CGRP is cardioprotective against heart failure. Our laboratory previously demonstrated that when α-CGRP KO mice underwent transverse aortic constriction (TAC) to develop pressure-overload heart failure, their survival rates were drastically decreased compared to the TAC-WT mice (Li et al., 2013). Additionally, the TAC-KO mice displayed a greater extent of dysfunctional cardiac remodeling and left ventricular hypertrophy, and they had reduced cardiac functions compared to the TAC-WT mice. Further, cardiomyocytes of the TAC-KO mice exhibited greater amounts of fibrosis, inflammation, and apoptosis, and had lower rates of angiogenesis as opposed to the TAC-WT mice. Our lab also showed that when we deliver native α-CGRP through osmotic mini-pumps for 28 days to the TAC-WT mice, CGRP administration is cardio-protective against pressure-overload induced heart failure (Kumar et al., 2019b). Our data showed that α-CGRP delivery preserved cardiac functions and inhibited left ventricular apoptosis, fibrosis, and cardiac hypertrophy in the TAC mice, and thus protecting hearts from pressure-induced heart failure. Based on our observations in this study, we proposed that TAC-induced pressure overload increased the nuclear level of sirt1 via the direct and/or indirect activation of AMPK and thus impaired mitochondrial function. As a result of mitochondrial dysfunction, there was a significantly higher number of apoptotic cells in the TAC-hearts due to larger amount of ROS produced. Administration of α-CGRP attenuated TAC-induced increased activation of sirt1 and AMPK, and inhibited oxidative stress and apoptotic cell death in TAC mice who received α-CGRP. Together, these events inhibited cardiac hypertrophy and protected hearts at the pathophysiological levels. These results suggest that α-CGRP might be a therapeutic agent to treat and prevent cardiac diseases. However, non-applicability of osmotic mini-pumps in humans and short half-life of α-CGRP in circulation (∼5.5 min in human plasma) limits the use of peptide (as the case with other peptides) in long-term treatment regime. To increase the bioavailability of peptide, we utilized an FDA approved alginate biomaterial as a drug carrier and developed an α-CGRP delivery system (Kumar et al., 2020). Alginate is an immunologically inactive natural biopolymer isolated from the seaweeds and has been used as a delivery vehicle for a number of biological materials, including cells, DNA, proteins, and peptides (Zhang et al., 2011; Moore et al., 2014; Annamalai et al., 2018; Gheorghita Puscaselu et al., 2020). α-CGRP was encapsulated into alginate polymer using an electrospray method and alginate-α-CGRP microcapsules of 200 μm diameter were prepared by passing a mixture of alginate and α-CGRP through a positively charged 30-G blunt end syringe needle at a constant flow rate (60 mm/h) under high voltage current (6 KV) into the 150 mM CaCl_2_ gelling solution. The distance between syringe needle and bathing soln was kept 7 mm (Kumar et al., 2020; Figure 5). α-CGRP-filled alginate microcapsules released peptide, most likely through diffusion, for an extended period (up to 6 days) where higher concentration was released in first few hours known as a “burst” and then constant but lower concentration for up to 6 days. Alginate-α-CGRP microcapsules were non-toxic to cardiac cells tested (rat H9C2 cardiac myoblast cell line and mouse HL-1 cardiac muscle cell line) in *in vitro* assays. The efficacy of these microcapsules was tested in the TAC-pressure overload mice. Alginate-α-CGRP microcapsules administered on day-2 or day-15 post-TAC attenuated cardiac hypertrophy and improved cardiac functions in the TAC-mice. Delivery of alginate-α-CGRP microcapsules lowered TAC-pressure induced LV apoptotic cell death, oxidative stress, and fibrosis in the TAC-alginate-CGRP group of mice. Alginate-α-CGRP microcapsules can be dehydrated and reconstituted in an buffered saline solution of distilled water. These capsules can be put in pluronic gels or collagen gels or injected as we have done either IP or sub cutaneious. Although α-CGRP release profile was performed in *in vitro* assays, the pharmacology and pharmacokinetics of released peptide in different organs and tissues under *in vivo* conditions are being investigated. The use of alginate is further enhanced due to it being an immunologically inactive polymer hence does not exhibit adverse effects *in vivo*. The cardioprotective effects of CGRP have been demonstrated in TAC-mice after administering CGRP encapsulated microcapsules via subcutaneous route. However, the oral route is a preferred method for delivery of most therapeutic agents, therefore the cardioprotective effects of alginate-α-CGRP microcapsules delivered through the oral route is of keen interest the lab. Furthermore, numerous additional benefits for using alginate microcapsules are: these microcapsules can be freeze-thawed without losing structural integrity; they can be stored at very low temperature, and they can be lyophilized. These studies indicate that our alginate-based delivery system is a potential approach to increase the bioavailability of CGRP in circulation and this therapeutic modality can be translated to treat cardiac diseases during long term treatment.

Several efforts are also being made in search of more stable bioactive α-CGRP-agonists to address peptide’s low bioavailability in human plasma (Nilsson et al., 2016). Aubdool et al., 2017, tested an acylated form of α-CGRP (half-life ∼7 h) in rodent models of Ang-II-induced hypertension and abdominal aortic constriction (AAC)-induced heart failure (Aubdool et al., 2017). Researchers demonstrated that subcutaneous administration of acylated-α-CGRP significantly reduced cardiac hypertrophy, fibrosis, and inflammation, and improved cardiac function in these disease rodent models. Recently, the cardioprotective effect of a metabolically stable CGRP analog, SAX, has been tested in a constant left anterior descending (LAD) occlusion rat model of acute myocardial infarction (Bentsen et al., 2021). Researchers reported that intraperitoneal administration of SAX (100 nmole/kg bwt per rat) improved myocardial recovery after myocardial infarction. These studies collectively suggest that utilization of α-CGRP (native or its analogs) and their delivery systems can be a potential strategy in preventing harmful cardiac remodeling and dysfunction present in cardiac diseases. As α-CGRP is a potent vasodilator and its higher level in plasma induces migraine, monitoring of blood pressure and migraine-like headache in humans is recommended while administering high doses of the peptide.

## Therapeutic Value of Alpha-Calcitonin Gene Related Peptide-Antagonists in Migraine Pain

Migraine is a chronic neurological disorder. Patients suffering from migraines show an increased level of CGRP in their saliva and plasma suggesting that CGRP plays an important role in the pathophysiology of migraine headache (Gallai et al., 1995; Cady et al., 2009; Hansen et al., 2010; Al-Hassany and Van Den Brink, 2020; Wattiez et al., 2020). It has been shown that the trigeminovascular system (containing the trigeminal ganglion and trigeminal nerves) becomes activated during a migraine episode that leads to the release of CGRP in the external jugular vein resulting in a migraine-like headache (Goadsby et al., 1988, 1990; Edvinsson, 2017). Recently developed CGRP-antagonists, which consist of humanized monoclonal antibodies that either bind to CGRP or its receptor, have been used to relieve the pain in migraine patients (Urits et al., 2019). These well tolerated and FDA-approved antibodies are erenumab, fremanezumab, galcanezumab, and eptinezumab, and are discussed below (Table 1).

**TABLE 1:** FDA approved anti-CGRP and anti-CGRP receptor antibodies for the treatment of migraine.

CGRP- antagonists	Manufacturer	Mode of action	Dose regime	Response in migraine patients	Potential side effects
Erenumab (Aimovig)	Amgen/Novartis	Blocks CGRP receptor	s.c., once monthly	Reduced monthly migraine days in patients with chronic and episodic migraines	Pain and redness at injection site, and constipation
Fremanezumab (Ajovy)	Teva Pharmaceuticals	Binds to α- and β- forms of CGRP	s.c., once monthly/quarterly	Reduced migraine days by an average of 5 days a month in patients with chronic migraine and reduced migraine days by an average of 3.5 days a month in patients with episodic migraine	Injection site reactions. May cause allergic reactions, such as itching, rash, and hives
Galcanezumab (Emgality)	Eli Lilly	Binds to α- and β- forms of CGRP	s.c., once monthly	Cut the number of monthly migraine days by 50% or more for some people	Injection site reactions. May cause allergic reactions, such as itching, rash, hives, and trouble breathing
Eptinezumab (Vyepti)	Lundbeck Seattle BioPharmaceuticals	Binds to α- and β- forms of CGRP	lntravenously (i.v.), once quarterly	Reduced monthly migraine days in patients with chronic and episodic migraines	Nasopharyngitis and hypersensitivity

### Erenumab (Aimovig)

Erenumab (commercial name Aimovig) is an FDA-approved fully monoclonal IgG2 antibody that selectively blocks the CGRP receptor, and subsequently downstream molecular signaling, to prevent the onset of migraines (Shi et al., 2016; Markham, 2018). It is administered once per month in patients to prevent the onset of migraines. Erenumab’s ability to attenuate migraine symptoms is primarily attributable to its ability to block CGRP receptors on the trigeminal ganglion and its branches. It is also thought that erenumab’s inhibitory actions on cerebral and meningeal blood vessel vasodilation plays a role in migraine treatment and prevention. Additionally, erenumab may block satellite cells from releasing inflammatory modulators and nitric oxide (NO) in response to α-CGRP exposure (Edvinsson et al., 2018).

A randomized, double-blind, and placebo-controlled phase II clinical trial showed that administration of 70 or 140 mg doses of erenumab to 667 chronic migraine patients reduced migraine days per month (Tepper et al., 2017). In a 6 month, randomized, double-blind, placebo-controlled phase III clinical trial (STRIVE trial), erenumab was injected subcutaneously monthly in doses of either 70 mg or 140 mg in 955 patients with episodic migraines to assess its safety and efficacy (Goadsby et al., 2017). Erenumab reduced the average number of migraine days per month by 43.3% (3.2 days) in patients given 70 mg doses and 50% (3.7 days) in patients injected with 140 mg doses, compared to a 26.6% reduction (1.8 days) in the placebo group.

An open-label, 5-year treatment phase followed 383 episodic migraine patients who had enrolled in a 12-week, double-blind, placebo-controlled clinical trial of erenumab (Ashina et al., 2021). These patients began the clinical trial receiving 70 mg doses of erenumab, but 250 patients were later switched to 140 mg doses. This open-label study tested the long-term safety and efficacy of erenumab by investigating changes in baseline monthly migraine days, monthly acute migraine-specific medication days, and health-related quality of life. By year 5 of erenumab use, these patients experienced an average reduction of 62.3% of monthly migraine days (–5.3 days per month), a –4.4 days reduction in acute migraine-specific days per month. This study also found that long-term erenumab use is not associated with increased frequency of adverse side effects. Therefore, the study concluded that erenumab is a safe and effective drug for migraine prevention.

### Fremanezumab (Ajovy)

Fremanezumab (TEV-48125) is a fully monoclonal IgG2 antibody that blocks CGRP signaling by binding to α- and β-forms of CGRP to inhibit these ligands from interacting with the CGRP receptor (Hoy, 2018). Approved by the FDA and developed by Teva Pharmaceuticals, fremanezumab (commercial name Ajovy) is administered monthly or quarterly via a subcutaneous injection to prevent migraine attacks (Friedman and Cohen, 2020). *In vitro* studies found that fremanezumab inhibits CGRP-induced vasodilation of intracranial and abdominal arteries. Fremanezumab is given in doses of 225, 675, or 900 mg, and its maximum concentration in plasma is apparent 5–7 days after administration, which indicates that it is absorbed slowly into the circulation. Fremanezumab has a plasma half-life of ∼31 days (Fiedler-Kelly et al., 2019).

A randomized, double-blind, placebo-controlled, parallel-group clinical trial tested the effectiveness of fremanezumab by administering 225 mg monthly or 675 mg quarterly to episodic migraine patients (Dodick et al., 2018). Patients in the 225 mg group experienced a reduction in mean migraine days per month from 8.9 to 4.9 days. The number of mean migraine days per month in subjects injected with 675 mg of fremanezumab quarterly dropped from 9.2 to 5.3 days, while those in the placebo group reported a reduction of 9.1 migraine days per month to 6.5 days.

Another randomized, double-blind, placebo-controlled, parallel-group study administered fremanezumab monthly or quarterly in chronic migraine patients (Silberstein et al., 2017). Patients who received fremanezumab monthly experienced a 4.6 days reduction of mean number of headache days per month, while those in the quarterly group reported a 4.3 days reduction. Both groups compared to the placebo were significant, as the reduction in number of headache days per month in the placebo group was 2.5 days. Additionally, 41% of patients in the monthly fremanezumab group and 38% in the quarterly group reported a reduction of ≥50% of headache days per month compared to 18% in the placebo group.

A 12-week, phase III clinical trial determined the efficacy of fremanezumab in patients with difficult-to-treat chronic or episodic migraines (Ferrari et al., 2019). Patients with difficult-to-treat migraines were defined by failure to respond to 2–4 of the following migraine preventative medications: beta-blockers, anticonvulsants, tricyclic antidepressants, calcium channel blockers, angiotensin II receptor antagonists, or onabotulinumtoxin A. In this randomized, double-blind, parallel-group study, patients received monthly or quarterly subcutaneous injections of either fremanezumab or a placebo. At the end of the 12 weeks, the study found that patients in both fremanezumab groups experienced a significant reduction in migraine days per month compared to the placebo. The monthly fremanezumab group reported an average of 4.1 fewer migraine days per month, while the quarterly fremanezumab patients experienced a 3.7-day reduction. Fremanezumab also reduced the frequency of monthly headache days of at least moderate severity in both groups (–4.2 days in monthly group; –3.9 days in quarterly group; –0.6 days in placebo group).

The HALO long-term study (LTS), a double-blind, randomized, parallel-group, 12-month, phase III clinical trial, investigated the long-term efficacy of fremanezumab in 1,890 patients with chronic or episodic migraines (Goadsby et al., 2020). At the end of the study, over 50% of patients with chronic migraine and ∼66% of patients with episodic migraine reported a ≥50% reduction in migraine days per month from their baseline levels. This investigation also found that as the study progressed, more patients reported reductions in monthly migraine days, indicating that fremanezumab became more effective with longer treatment duration.

### Galcanezumab (Emgality)

Developed by Eli Lilly & Co., galcanezumab (commercial name Emgality) is an FDA-approved humanized monoclonal antibody used for migraine and cluster headache prevention (Lamb, 2018). Galcanezumab has an apparent half-life of ∼27 days in serum and binds to the CGRP ligand to prevent interaction with the CGRP receptor, thus abolishing the biological activity of α-CGRP (Dodick et al., 2014b). Healthy volunteers injected with a single dose of 75–600 mg of galcanezumab experienced significantly more inhibition of capsaicin-induced dermal blood flow compared to a placebo group at all post-dose time intervals (days 3, 14, 28, and 42). Galcanezumab is administered monthly via subcutaneous injection, and it is slowly absorbed into circulation, as it reaches maximum plasma concentration ∼5 days post-injection.

In a clinical trial (EVOLVE), the safety and efficacy of galcanezumab was evaluated in episodic migraine patients treated with 120 mg or 240 mg galcanezumab or a placebo group (Stauffer et al., 2018). By the end of the 6-month period, galcanezumab reduced the number of monthly migraine days by 4.7 and 4.6 days at doses 120 and 240 mg, respectively. Similarly, 65% of patients taking galcanezumab reported a > 50% reduction of mean headache days per month compared to 42% in the placebo group.

A randomized, double-blind, placebo-controlled phase III clinical trial (REGAIN) investigated the efficacy of galcanezumab by injecting monthly doses of 120 mg (with an initial 240 mg loading dose) or 240 mg of galcanezumab or a placebo in chronic migraine patients (Detke et al., 2018). Patients in the 120 mg galcanezumab group experienced a mean reduction in number of headache days per month of –4.8 days. Similarly, the 240 mg galcanezumab group reported a –4.6 day reduction, while patients in the placebo group experienced a –2.7 day reduction in number of headache days per month.

In addition, an 8-week, randomized, double-blind, placebo-controlled phase III clinical trial injected 300 mg doses of galcanezumab once per month in 106 adults with episodic cluster headaches (Goadsby et al., 2019). During weeks 1–3, galcanezumab decreased the number of weekly cluster headaches by 8.7, as opposed to 5.2 in the placebo group. Additionally, by week 3, 71% of patients receiving galcanezumab reported a ≥50% decrease in the incidence of weekly cluster headaches, compared to 53% in the placebo group.

### Eptinezumab (Vyepti)

Eptinezumab is a humanized monoclonal antibody that is used to prevent the onset of migraine attacks (Dodick et al., 2014a; Baker et al., 2020). Developed by Lundbeck Seattle BioPharmaceuticals under the commercial name Vyepti, eptinezumab binds to α- and β-forms of CGRP to prevent ligand-receptor interaction, and therefore blocks CGRP signaling. Eptinezumab has a half-life of ∼27 days, and it achieves steady state serum concentrations following its initial administration and subsequentially after each infusion once every 3 months.

PROMISE-1, a randomized, double-blind, multicenter, placebo-controlled phase III clinical trial assessed the efficacy and tolerability of eptinezumab by intravenously administering 30, 100, or 300 mg doses of eptinezumab or a placebo every 3 months for 12 months in 888 adults with episodic migraines (Ashina et al., 2020; Smith et al., 2020). Patients who received 100 mg doses of eptinezumab reported a 3.9 day reduction in monthly migraine days during weeks 1–12, compared to 3.2 days in the placebo group. Similarly, patients who were given 300 mg of eptinezumab experienced a 4.3 day decrease in monthly migraine days during weeks 1–12. Because the 30 mg dose of eptinezumab failed to be statistically significant on the primary efficacy end point, this dosing regimen was not approved by the FDA. Throughout weeks 1–4, the 100 mg and 300 mg eptinezumab groups had significantly higher rates of ≥75% reductions in monthly migraine days from baseline compared to the placebo (31% in 100 mg group, 32% in 300 mg group, and 20% in placebo). During weeks 1–12, significantly more patients who received 300 mg doses of eptinezumab reported ≥50% decrease in monthly migraine days vs. placebo (56 vs. 37%). Additionally, 30% of 300 mg eptinezumab patients reported a ≥75% reduction in monthly migraine days during weeks 1–12, compared to 16% in the placebo group. All eptinezumab dosing regimens decreased the frequency of monthly migraine days compared to the placebo through week 48, and the beneficial effects of eptinezumab persisted throughout the entire study. The number of patients who experienced a ≥50% or a ≥75% reduction in monthly migraine days were significantly higher in those who received 100 or 300 mg doses of eptinezumab compared to the placebo from weeks 1 to 48. By week 48, the average reduction in monthly migraine days was –4.5 and –5.3 days in the 100 mg and 300 mg eptinezumab groups, compared to –4.0 days in the placebo group. Of all the patients treated with eptinezumab, ∼70% experienced a ≥50% migraine response during at least 6 months of the study and >20% reported ≥50% migraine response during all 12 months of the study.

Moreover, a large randomized, placebo-controlled, double-blind, multicenter, phase III clinical trial (PROMISE-2) investigated the safety and efficacy of eptinezumab in 1,072 patients with chronic migraines (Lipton et al., 2020). Patients were randomized to an eptinezumab or a placebo group and received either 100 or 300 mg intravenous doses every 3 months for 6 months. The effects of eptinezumab were apparent after the first day of dosing, as 28.6% of patients who received 100 mg of eptinezumab and 27.8% of patients administered 300 mg of eptinezumab reported having a migraine after the first day of dosing compared to 42.3% of patients in the placebo group. Eptinezumab significantly reduced the frequency of monthly migraine days during weeks 1–12 in both the 100 mg (–7.7 days) and 300 mg (–8.2 days) groups compared to the placebo (–5.6 days). During weeks 1–12, 26.7% of patients who received 100 mg doses of eptinezumab and 33.1% of the 300 mg eptinezumab group reported ≥75% reduction rates in monthly migraine days, compared to 15% in the placebo group. The most common side effect of eptinezumab use was nasopharyngitis, which occurred in 9.4% of patients who received 300 mg of eptinezumab, compared to 6.0% of patients in the placebo group. Less than 1% of patients (7 from eptinezumab group and 3 from placebo group) experienced a serious treatment emergent adverse effect (TEAE), including nervous system disorders, injury, poisoning, and procedural complications. Based on these results, eptinezumab was determined to be a well-tolerated and effective medication for migraine prevention.

## Conclusion

Numerous biological, pharmacological, and genetic studies carried out in a variety of animal models have established the role of α-CGRP in normal and disease states, particularly in cardiovascular disease and migraine pain. In recent years the development of CGRP-antagonists and their successful use in humans to treat/prevent migraine-like headache is remarkable. Growing evidence suggest that, in addition to classical CGRP-receptor, CGRP can interact with other CGRP-responsive receptors (such as AMY1 receptor) present on the cell surface, hence studying the pharmacological effects of developing CGRP-agonists/-antagonists on these receptor subtypes might be valuable for the successful development of CGRP-based therapy for diseases. Moreover, the ongoing efforts to enhance the bioactivity of CGRP in circulation via CGRP-analogs and delivery systems are promising therapeutic agents to treat patients suffering from cardiovascular diseases. The alginate-based delivery system for α-CGRP is showing promising results to increase the bioavailability of the peptide and protect against pressure-induced heart failure. Further testing of this delivery system is needed in other rodent models of cardiovascular diseases, including hypertension, myocardial infarction, ischemia-reperfusion injury, and also other modes of alginate-CGRP microcapsules delivery is needed to verify in these cardiac diseases. These endeavors are a hopeful avenue for CGRP-based cardiovascular therapeutics in the coming years. Another aspect that is needed to consider in CGRP biology is the accurate measurement of peptide in the biological samples. α-CGRP is a very short-lived peptide making it difficult to measure accurately in samples. Although radio-immunoassay and enzyme-immunoassay are employed, more sensitive detection methods such as high-performance liquid chromatography (HPLC) and mass spectroscopy should be considered to include in the CGRP measurement assays. It is also worthy to mention here that as α-CGRP has an important role in regulating regional organ blood flow in normal and pathophysiological conditions, it might be possible that migraine patients treated with CGRP-antagonists, that block CGRP-signaling, develop adverse side effects on heart and blood vessels. Hence long-term follow-up of these patients in terms of cardiovascular safety is advisable.

## Author Contributions

JP was responsible for the design and analysis of the data and construction and editing of the manuscript. DD was responsible for the design, analysis and construction, and editing of the manuscript. AK responsible for the generation of TAC animals and data presented in the manuscript, as well as contributed to the writing of the manuscript. MW was responsible for the generation of data presented in the manuscript, in addition to editing the manuscript. AH was responsible for the generation of data presented in the manuscript, in addition to editing the manuscript. All authors contributed to the article and approved the submitted version.

## Conflict of Interest

The authors declare that the research was conducted in the absence of any commercial or financial relationships that could be construed as a potential conflict of interest.

## Publisher’s Note

All claims expressed in this article are solely those of the authors and do not necessarily represent those of their affiliated organizations, or those of the publisher, the editors and the reviewers. Any product that may be evaluated in this article, or claim that may be made by its manufacturer, is not guaranteed or endorsed by the publisher.

## Abbreviations

Ang-II: Angiotensin-II
α-CGRP: Alpha-calcitonin gene-related peptide
DRG: Dorsal root ganglia
I/R injury: Ischemia/reperfusion injury
KO: Knock-out
NO: Nitric oxide
NOS: Nitric oxide synthase
RAAS: Renin angiotensin aldosterone system
RAMP: Receptor activity modifying protein
ROS: Reactive oxygen species
SP: Substance P
TAC: Transverse aortic constriction
WT: Wild-type.
